# Deep Learning-Based Evaluation of Ultrasound Images for Benign Skin Tumors

**DOI:** 10.3390/s23177374

**Published:** 2023-08-24

**Authors:** Hyunwoo Lee, Yerin Lee, Seung-Won Jung, Solam Lee, Byungho Oh, Sejung Yang

**Affiliations:** 1 Department of Biomedical Engineering, Yonsei University, Wonju 26493, Republic of Korea; hulee6239@yonsei.ac.kr; 2 Department of Precision Medicine, Yonsei University Wonju College of Medicine, Wonju 26426, Republic of Korea; leey01@yonsei.ac.kr; 3 Department of Dermatology, Yonsei University Wonju College of Medicine, Wonju 26426, Republic of Korea; jsw0826@yonsei.ac.kr (S.-W.J.); solam@yonsei.ac.kr (S.L.); 4 Department of Dermatology, Cutaneous Biology Research Institute, Yonsei University College of Medicine, Seoul 03722, Republic of Korea

**Keywords:** convolutional neural network, ultrasound image, benign skin tumor, class activation map

## Abstract

In this study, a combined convolutional neural network for the diagnosis of three benign skin tumors was designed, and its effectiveness was verified through quantitative and statistical analysis. To this end, 698 sonographic images were taken and diagnosed at the Department of Dermatology at Severance Hospital in Seoul, Korea, between 10 November 2017 and 17 January 2020. Through an empirical process, a convolutional neural network combining two structures, which consist of a residual structure and an attention-gated structure, was designed. Five-fold cross-validation was applied, and the train set for each fold was augmented by the Fast AutoAugment technique. As a result of training, for three benign skin tumors, an average accuracy of 95.87%, an average sensitivity of 90.10%, and an average specificity of 96.23% were derived. Also, through statistical analysis using a class activation map and physicians’ findings, it was found that the judgment criteria of physicians and the trained combined convolutional neural network were similar. This study suggests that the model designed and trained in this study can be a diagnostic aid to assist physicians and enable more efficient and accurate diagnoses.

## 1. Introduction

Ultrasound is an attractive modality for the noninvasive evaluation of subcutaneous lesions because it is simple to use, safe, and relatively inexpensive [1,2,3,4]. In the field of cutaneous oncology, an ultrasound examination performed before surgery can provide information about tumor type and size, locate the existence of surrounding vessels, identify the best location for the incision, and set the range while viewing the ultrasound screen in real-time with the patient [5,6,7].

However, it is difficult to apply ultrasound imaging to diagnosis owing to limitations such as noise, artifacts, and complexity [8]. In the ultrasonic image, unlike in other fields, there are additional acoustic noise and speckle noise, which can make it more difficult to extract features from the ultrasound image. Moreover, various artifacts occur due to assumptions such as consistency of speed of sound, beam axis straightness, attenuation consistency in tissue, and pulse only going to the target. Furthermore, the anatomical complexity of the human body is added, the more difficult it is to interpret the image information [9]. Therefore, physicians often make a diagnosis through a specific biopsy rather than simply making a diagnosis on ultrasound images.

Recently, convolutional neural networks (CNNs), which are one of the artificial intelligence fields, have been actively applied to various medical vision modalities, including ultrasound imaging [10]. It is also widely used in the diagnosis of skin lesions in ultrasound imaging [11,12,13,14,15,16,17,18]. The CNNs used in these studies produced expert-level diagnostic accuracy compared to histopathological results. Also, since it is a real-time level of diagnostic speed, it can serve as an excellent diagnostic aid.

Therefore, we believe that the deep-learning model can achieve accurate predictions and classifications of various skin diseases based on ultrasound images. In this study, using combined CNN, we designed an automatic ultrasound image diagnosis algorithm and applied it to three benign skin tumors (BSTs), including epidermal cyst, the most common cutaneous cyst; lipoma, the most common benign soft-tissue neoplasm; and pilomatricoma, the most common appendage tumor in children [19]. The proportion of the three tumors has been surveyed to account for approximately 70% of BSTs in South Korea [20]. Then statistical analysis was also performed on the trained combined CNN’s prediction using class activation map (CAM) [21] and physicians’ findings.

## 2. Materials and Methods

### 2.1. Study Subjects

The institutional review board (IRB) of Yonsei Severance Hospital (Approval Number: 4-2020-0910) approved this retrospective, single-center study, and the written informed consent of patients was waived. All research was performed in accordance with relevant guidelines and regulations.

The images for datasets were taken and diagnosed at the Department of Dermatology at Severance Hospital in Seoul, Korea, between 10 November 2017 and 17 January 2020. In addition, sonographic imaging was performed using a Noblus ultrasound system (Hitachi, Inc., Tokyo, Japan) with two probes (7–13 and 5–18 MHz). The inclusion criteria for this study were patients who underwent surgery for benign skin tumors and received ultrasound examination. On the other hand, the exclusion criteria involved patients who did not undergo ultrasound examination or did not have skin pathology testing performed on the excised tissues after surgery.

**Figure 1:**
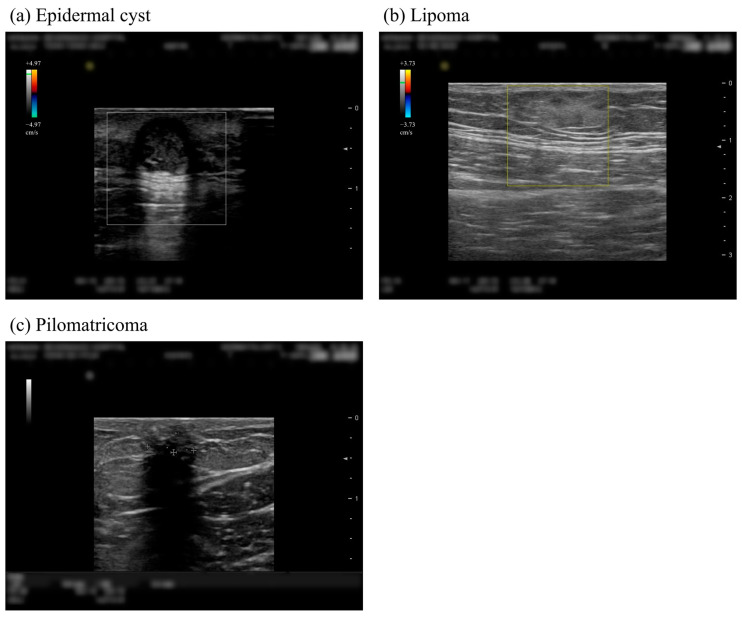
Example images of three skin lesions correctly classified by the convolutional neural network algorithm. The various symbols in the figure are for the services provided by the ultrasound imaging equipment, including the measurement of lesion size. (**a**) Sample image of a patient with epidermal cyst. Our algorithm predicts this image with 73.85%, 9.97%, and 16.18% probability rates for three classes (epidermal cyst, lipoma, pilomatricoma) in order. (**b**) Sample image of a patient with lipoma. Our algorithm predicts this image with 6.76%, 77.11%, and 16.12% probability rates for three classes (epidermal cyst, lipoma, pilomatricoma) in order. (**c**) Sample image of a patient with pilomatricoma. Our algorithm predicts this image with 13.93%, 10.50%, and 75.56% probability rates for three classes (epidermal cyst, lipoma, pilomatricoma) in order.

The dataset consists of 698 images from 250 patients; mean [SD] age of 46.4 [16.6] years; 156 men (62.4%); 149 patients with epidermal cysts, 74 patients with lipoma, and 32 patients with pilomatricoma. Additionally, there were 4 patients who received diagnoses of both epidermal cysts and lipoma and 1 patient who received diagnoses of both pilomatricoma and lipoma. Each patient’s benign tumor type was determined by histopathological examination through biopsy. The example images of each benign skin tumor can be seen in Figure 1.

### 2.2. Data Preprocessing

The entire original data contains a number of Doppler images to identify the characteristics of blood flow. In the Doppler-colored region of the images, the morphological characteristics of benign tumors would be partially damaged, which may cause performance degradation when performing deep learning. Therefore, it is necessary to fill the corresponding colored areas using image processing method. In this study, in order to fill in the areas, a traditional inpainting technique that fills the empty areas using the surrounding information of the original image was used [22]. In addition, all the outer parts other than the skin area displayed in the center of the original image are all black with a value of 0, so it is unnecessary information. Therefore, for all data sets, the middle skin areas are all cropped to be used as dataset.

Data augmentation is a method of increasing the amount of data by acquiring new data through image transformation in an existing data set. There are dozens of ways to transform an image, including geometric methods such as translate and rotate, methods that transform pixel values such as invert, solarize, and equalize, and methods of adding noise such as Gaussian and speckle. However, it takes a lot of effort and time to select the most suitable transformation methods for the given data because it requires many trials, including deep-learning model training. Therefore, in this study, Fast AutoAugment [23], one of state-of-the-art data augmentation methods that automatically finds transformation methods suitable for a given data set, was applied. Through this, it was possible to compensate for the performance degradation of the deep learning model caused by the small number of data sets.

### 2.3. Combined CNN Structure

**Figure 2:**
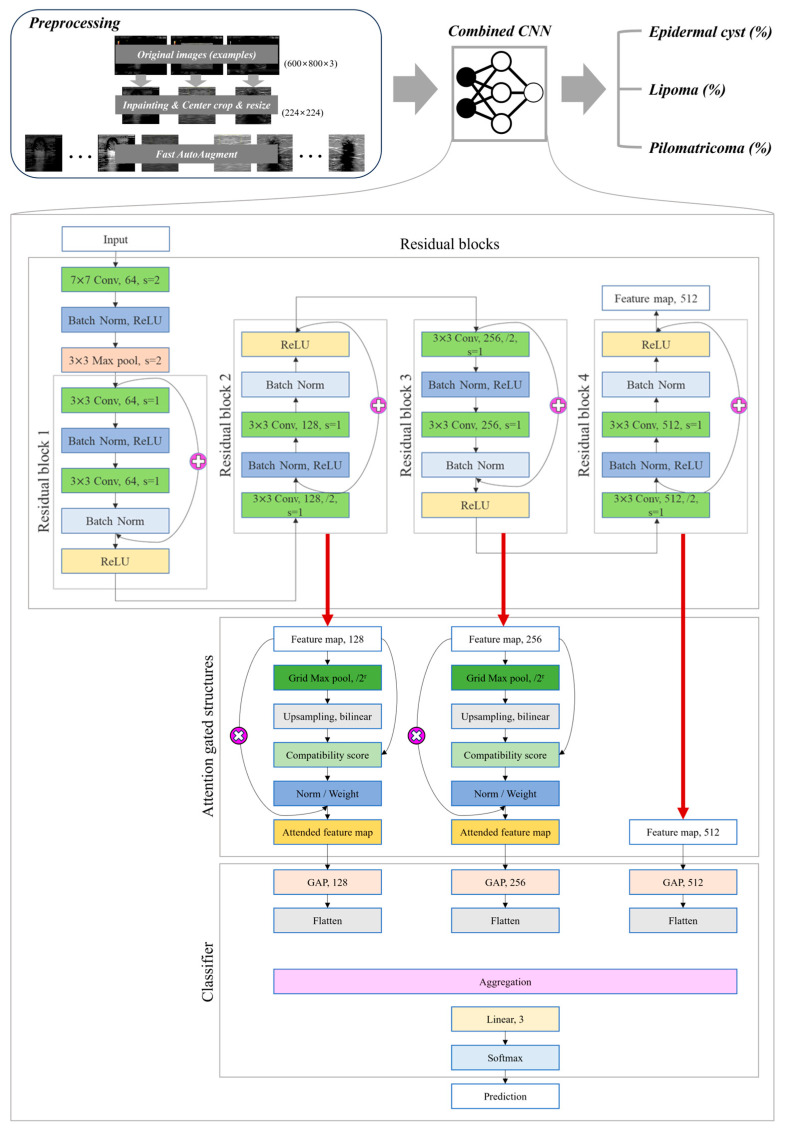
Overall flow of the designed algorithm. In residual blocks, the plus sign represents the process of adding the output of the previous layer to the output of the batch normalization block, while in attention gated structures, the multiplication sign represents the element-wise multiplication of the feature map and the attention map.

Two CNN model structures were combined to produce a new CNN model suitable for the current data set and task (Figure 2). The first structure is a residual structure [24] that has been used to achieve high performance in various tasks since the publication of the paper. The second is attention-gated structure [25], one of the state-of-the-arts in the classification field in ultrasound images. Compared with training using only residual structure, higher performance was obtained when training by combining attention-gated structure. The output of the combined CNN is generated as probability values for three benign tumors.

#### 2.3.1. Residual Structures

For the residual structure in the combined CNN, the structure up to the 4th residual block in the pretrained ResNet18 architecture was used. By inputting the feature maps in blocks 2 and 3 into the attention gate, each new feature map is obtained. Then, the feature map of block 4 and the two newly obtained feature maps are flattened and aggregated after passing through a fully connected layer to obtain the final output. The output obtained in this way finally goes through the softmax activation function and returns a probability value for each benign skin tumor.

#### 2.3.2. Attention-Gated Structures

**Figure 3:**
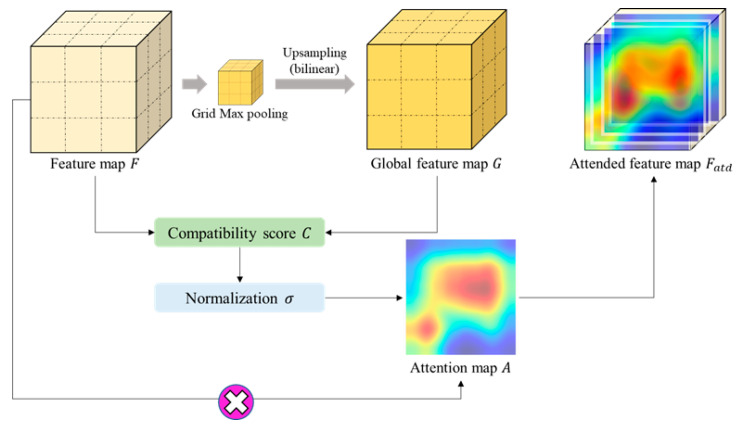
Overview of the attention feature map generation. The multiplication sign represents the element-wise multiplication of the feature map and the attention map.

The overall flow of the attention-gated structure is shown in Figure 3. First, a compatibility score is calculated using the current feature map and the global feature map, and the calculated compatibility score is normalized. The map created at this time is called an attention map. Finally, the output of the attention unit is completed through element-wise multiplication of the calculated feature map and the attention map. The equation for computation of the attention map is as follows:

(1) $$A\left(F^{l},G\right)=W_{c}\sigma \left(W_{f}f_{i}^{l}+W_{g}g_{i}+b_{g}\right)+b_{c}\;where,\;\sigma =e^{C_{l}^{i}}/\overset{I}{\underset{i=1}{\sum }}e^{C_{l}^{i}}$$

First, $F^{l}$ means the feature maps of the current layer l, and G becomes a global feature for the feature map. On the right side of the equation, $W_{c}$, $W_{f}$ and $W_{g}$ all mean weights, which are trainable parameters, and are weights for compatibility, feature map, and global feature map, respectively. Then, in order to match the dimensions of the feature map and the global map, each weight is multiplied and then the sum of the two maps is calculated. Through this process, the compatibility map $c=W_{f}f_{i}^{l}+W_{g}\;g+b_{g}$ is completed. Then, normalization is performed using the softmax function σ. Multiplying the result by the weight once again and adding the bias, the attention map for the current feature map is completed. By performing element-wise multiplication of the completed attention map with the original feature map, the final attended feature map is obtained (Equation (2))

(2) $$F_{atd}\left(F^{l}\right)=A\left(F^{l},\;G\right)\cdot F^{l}$$

Performing global average pooling and propagating the completed attended feature map separately from the existing feature map is done by an attention-gated structure. This structure is robust to the final classification result because it adds critical information to the feature maps for each layer.

As shown in Figure 3, a global feature map is created through grid max pooling. Unlike methods that converge to a single value, such as global average pooling (GAP) or global max pooling (GMP), since max pooling for each grid of a specific size is used, local information can be preserved in a given feature map. In general, medical imaging images have extremely local features, and thus using grid max pooling can improve the performance of deep-learning models.

### 2.4. Optimizing Combined CNN

Optimization of combined CNN was performed on an NVIDIA RTX 2080Ti graphics processing unit (GPU) 11 GB. The development environment of deep-learning algorithm is Python 3.9.5 using pytorch 1.7.1 with CUDA 10.1.

#### 2.4.1. Data Preparation

For five-fold cross-validation, patients were divided into five groups of 50 each; one group was set as a test set, and the other groups were set as a training set. In addition, as a result of Fast AutoAugment, sub-policies consisting of image transformation methods, probability, and magnitude values were determined, and data augmentation was performed for each train set. The detail of Fast AutoAugment algorithm is introduced in Supplementary Materials. By data augmentation, the number of train sets increased by twenty-one times, and the overall data distribution can be seen in Table 1.

#### 2.4.2. Training Details

When training the designed combined CNN, the optimum hyperparameters were determined by multiple experiential processes. The hyperparameters determined are as follows: first, the focal loss is used as the loss function, the alpha value is set to 0.25, and the gamma value is set to 3. For the optimizer, adaptive moment estimation (Adam) [26] was used with learning rate of 1 × 10^−4^ (β_1 = 0.9 and β_2 = 0.999). In addition, a scheduler that sets the learning rate to decrease by 0.1 for every 7th epoch was used. The early stopping technique, which stops training if it is not optimized above a certain level, is used, and the model weights values of the epoch, which had the best performance, are adopted. The input image size was set to 224 × 224 according to the cited structure, and the mini-batch size was set to 64, which pooled the best performance among the multipliers of 2 that did not exceed the GPU capacity. Five-fold cross-validation was performed, and evaluation of each fold was performed based on confusion matrix.

### 2.5. Statistical Analysis

Statistical analyzes were performed to determine whether the judgment criteria of physicians and the judgment criteria of the trained CNN model were similar. First, the thresholded CAM images for all data are extracted in an overlapping form with the original images. After showing the CAM images to the physician, the degree of how much the judgment criteria were matched for each data was assigned a level from 0 to 2. In this study, the level distribution of all data created was called decision consistency. Further, when diagnosing three benign skin tumors on ultrasound skin images, the sonographic features that physicians use as a standard are shown in Table S1 in Supplementary Materials. During the analysis, the clinicians were blinded to the categories of benign skin tumors when reviewing the CAM images. This blinding was performed to ensure unbiased evaluation and minimize any potential bias that could affect the results.

The decision consistency made with levels 0, 1, and 2 is compared with the confidence distribution of the trained model for statistical analysis. In this case, the confidence distribution is made with the distribution of probability values for the corresponding classes when the trained model makes predictions. Categorical variables were presented as the number and proportion of patients and were compared using the Pearson chi-square test. Continuous variables are presented as mean and were analyzed using two-way analysis of variance (ANOVA). Two statistical analyses consisting of linear-by-linear association and ANOVA tests were performed on these two distributions. Two statistical analyses were done at the 95% confidence interval, so it would be statistically significant if the *p*-value in each case was less than 0.05. This means that the more the trained model makes a judgment with high confidence, the more similar the focus on the image is to the physician’s. In other words, the designed model was trained to diagnose three benign skin tumors with criteria similar to physicians’ judgment. The IBM SPSS software version 25 was used for statistical analysis.

## 3. Results

### 3.1. Quantitative Evaluation

**Figure 4:**
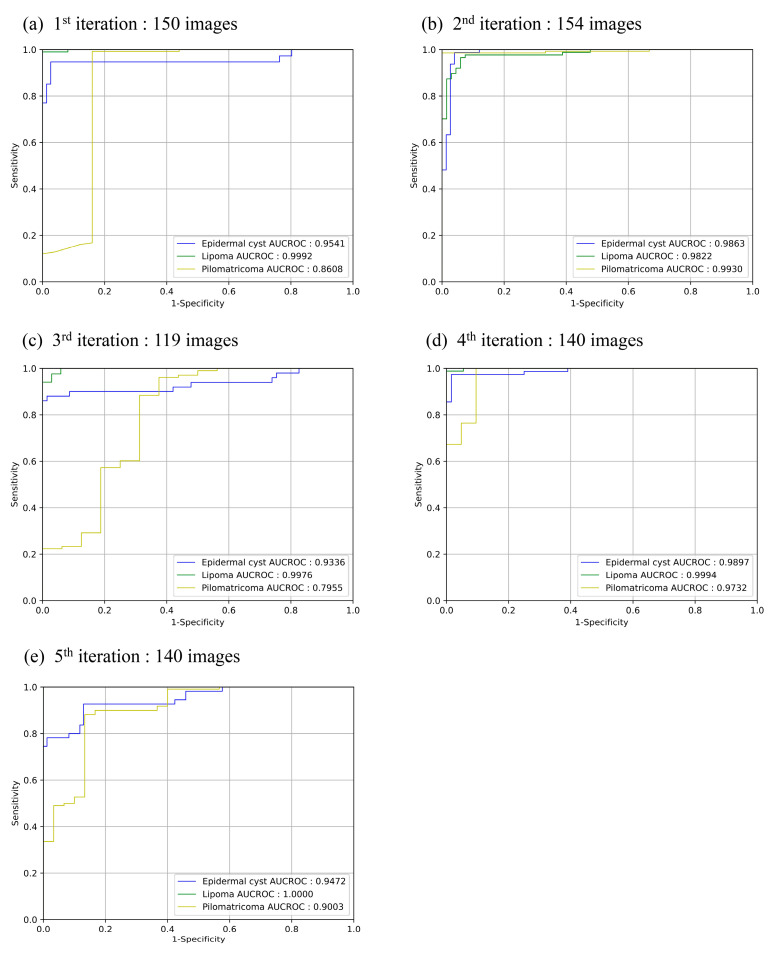
Receiver operating characteristic (ROC) curve for the test set for each iteration in cross-validation. In each graph, the curves and area under the receiver operating characteristic curve (AUROC) values for the three classes are displayed. Subfigures (**a**–**e**) illustrate the ROC curves for the first to fifth fold of the dataset.

Table 2 and Figure 4. Show the five-fold cross-validation results for 698 total ultrasound skin images. Figure 4 shows the plots of the receiver operating characteristic (ROC) curves for the test set with each iteration through cross-validation results. Table 2 shows a summary of the cross-validation results of the test set over five iterations specified for each BST. The classifier demonstrated excellent performance for classifying all three types of BST, exceeding 90% in nearly all metrics. However, the F1 score and sensitivity for pilomatricoma remained at around 70%.

### 3.2. Statistical Analysis

**Figure 5:**
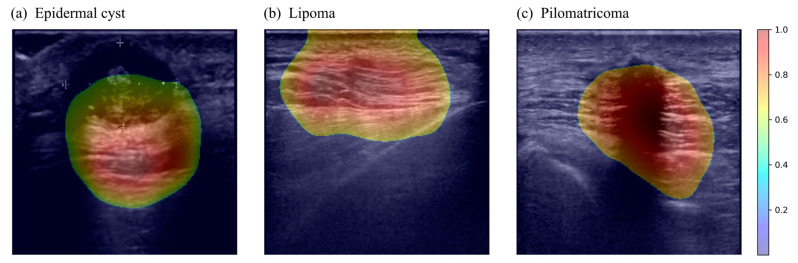
Example of the class activation map for each benign tumor of the trained model. Among the images accurately predicted by the trained model, they are examples of class activation maps corresponding to (**a**) epidermal cyst, (**b**) lipoma, and (**c**) pilomatricoma in order from left column. The color bar on the right side indicates the normalized class activation map value corresponding to each image.

When a deep learning model predicts, CAM visually shows which parts of a given image are concerned. The examples of the CAMs extracted from trained combined CNN are shown in Figure 5. Through the CAMs in the figure, it can be visually confirmed that the trained combined CNN is well activating the expression location of each benign skin tumor. The results of statistical analysis between the consistency distribution made by comparing physicians’ findings with CAMs and the confidence distribution of the trained model are as follows. First, as a result of the linear-by-linear association test, a *p*-value of <0.001 was obtained. Second, as a result of the ANOVA test, there was a significant difference in the confidence score between the three decision consistency groups (*p* < 0.001). Additionally, in the pairwise comparisons, the confidence scores in the all-matched cases were significantly greater than those in the case with non-matched (*p* < 0.001) or partially matched (*p* < 0.001) after the Bonferroni correction. The box plot and combined matrix for statistical analysis are shown in the Supplementary Materials.

## 4. Discussion

Over the past years, the development of ultrasound imaging technology has been in the spotlight as a first-line screening modality by enabling real-time observation of the location, size, and shape of skin tumors. Particularly in the dermatologic surgeon’s position, it has the advantage of notifying the existence of surrounding vessels before surgery, identifying the best location for the incision, and setting the range while viewing the ultrasound screen in real-time with the patient [6]. It is known that, particularly in cases like epidermal cysts, the risk of cystic rupture can lead to irregular shapes and potentially increase peripheral blood flow [5,27,28]. Furthermore, in conditions like pilomatricoma, the significance of detecting calcification has been proven, where ultrasound excels in identification [5,6]. After surgery, it can also help the surgeon evaluate whether the tumor is completely removed [7]. However, a suitable and reliable diagnosis of soft-tissue tumors requires long-term training for inexperienced physicians [29]. To solve this problem, a computer-aided diagnosis (CAD) system performed by extracting and analyzing morphologic or texture features was introduced, and the results were reported to be equivalent to the radiologists’ evaluation [30].

Recently, studies on the diagnosis of skin disease by analyzing skin images using artificial intelligence such as CNN have been actively conducted [31,32,33,34,35,36,37]. In particular, when applied to dermoscopic images, accuracy increases and significantly supports physicians’ decisions [38,39,40]. Unlike the CAD system, the diagnostic method using deep learning, as in this study, combines the two stages of feature extraction and classification into one, and the entire classification process is automated. In addition, it is advantageous in terms of time and labor because the features are automatically extracted and classified regardless of tumor type. However, to gain expert confidence in the diagnosis result of deep learning, it is necessary to analyze which features the model considers. Because the features are directly determined in CAD, this secondary work is not necessary. This can be a disadvantage of the diagnostic method using deep learning. However, if it is continuously proved that the deep learning model showed a high level of diagnostic results based on meaningful features, as in this study, it can be expected that such shortcomings will gradually disappear.

Nevertheless, there are few studies that analyze ultrasound images using artificial intelligence and apply them to diagnosis. The reason for such few publications may be due to the rare occurrence, but various types of soft-tissue tumors have limited the accumulation of results in a clinical database [29]. In addition, the ultrasound image is a device capable of intervening subjective factors in the inspection, such as changes or artifacts in the image depending on the pressure, direction, and position of the inspector pressing the probe.

The findings of this study show that our diagnosis algorithm using combined CNN showed high performance of diagnosis based on the histopathologic results of three benign tumors. Specifically, the model achieved a classification accuracy of 94.9, 98.2, and 94.5 for epidermal cysts, lipoma, and pilomatricoma. However, the F1-score and sensitivity for classifying pilomatricoma exhibited limited performance, primarily attributed to the restricted representation caused by insufficient data compared to the other two classes. In addition, we conducted three statistical tests, and the results revealed p-values below 0.001. It was found that the judgment area of physicians for the diagnosis from ultrasound images and that of the CNN classifier expressed in the CAM were significantly correlated. These statistical findings provide additional evidence supporting the reliability and precision of our CNN-based classifier in correctly pinpointing the regions of interest for diagnosis. Similar results have been shown in other ultrasonography of thyroid [41,42], liver [43], breast cancer [44,45], lymph node [46], fetal brain [47], and chest ultrasound diagnoses for Coronavirus disease (COVID-19) [48].

In contrast to many other clinical departments, dermatology is a unique department, benefiting from the visible accessibility of the skin, which allows for feasible examination and diagnosis. Furthermore, it is widely recognized that ultrasonography in dermatology has predominantly been employed for the assessment of malignant skin cancers, such as basal cell carcinoma, squamous cell carcinoma, and melanoma [49]. Consequently, there has been a lack of extensive use of ultrasonography for benign skin tumors, while these benign tumors constitute approximately 70% of benign skin tumors, making them of significant clinical importance. As a faithful diagnostic aid, showing the activation site in real-time is expected to help doctors identify areas to see and compliment areas that may be overlooked. The improvement in the accuracy of automatic diagnosis is expected to exceed its value as an auxiliary diagnostic tool. 

However, there are some limitations to the retrospective design of this study. Because the number of image data collected for each disease is nonuniform, there is a limit to comparative analysis. Likewise, it is expected that the accuracy of this algorithm may be lowered when other rare tumors are added. Another limitation is that this study included only sonographic images taken with a single model. An additional train with a larger dataset is required to achieve consistent performance for images taken in different environments. Moreover, a prospective clinical trial is required to investigate the efficacy of its usage in real clinical settings and improve patient outcomes. Therefore, in order to conduct a prospective clinical trial, a web service for the model trained in this study was developed, and a demo video was produced for it (Supplementary Materials). 

## 5. Conclusions

Ultrasound imaging technology is widely utilized in skin tumor examination and surgical support. At the same time, CAD systems based on skin images have been extensively developed, but a technology for diagnosing skin tumors automatically has been lacking. In this study, we created a CNN-based classifier to automate the diagnosis of skin tumors and revealed a significant correlation between the assessments of physicians and the results of the CNN. However, there were limitations that required validation and improvement of the efficacy of the model in real clinical settings with a larger dataset. This research indicates the potential for future automation of skin tumor diagnosis and furthermore, the possibility of applying learned features of skin diseases to large-scale disease understanding systems.

## Figures and Tables

**Table 1:** Data distribution for each fold of the five-fold cross-validation dataset. This table summarizes the number of images of training set and test set for each iteration of five-fold cross-validation and the number and ratio of the three classes in each dataset.

Dataset	Total No. of Images	Images, No. (%)		
	(Augmented ^a^)	Epidermal Cyst	Lipoma	Pilomatricoma
1st fold				
Training set	548 (11,508)	310 (56.6)	183 (33.4)	55 (10.0)
Test set	150	76 (50.7)	49 (32.7)	25 (16.7)
2nd fold				
Training set	544 (11,424)	311 (57.2)	165 (30.3)	68 (12.5)
Test set	154	75 (48.7)	67 (43.5)	12 (7.8)
3rd fold				
Training set	579 (12,159)	317 (54.7)	198 (34.2)	64 (11.1)
Test set	119	69 (58.0)	34 (28.6)	16 (13.4)
4th fold				
Training set	558 (11,718)	322 (57.7)	177 (31.7)	59 (10.6)
Test set	140	64 (45.7)	55 (39.2)	21 (15.0)
5th fold				
Training set	558 (11,718)	301 (53.9)	207 (37.1)	50 (9.0)
Test set	140	85 (60.7)	25 (17.9)	30 (21.4)

^a^ Fast AutoAugment method was used for augmentation only to the training dataset.

**Table 2:** Summary of the five-fold cross-validation result. This table shows the results for each benign skin tumor for five-fold cross-validation. Abbreviations: AUROC, area under the receiver operating characteristic curve; CI, confidence interval.

Tumor Types	Accuracy, % Mean ^a^ (95% CI ^b^)	AUROC, Mean ^a^ (95% CI ^b^)	F1 Score, Mean ^a^ (95% CI ^b^)	Sensitivity, Mean ^a^ (95% CI ^b^)	Specificity, Mean ^a^ (95% CI ^b^)
Epidermal cyst	94.9 (91.3–98.5)	0.962 (0.931–0.993)	95.5 (93.2–97.9)	97.9 (95.4–100.0)	92.4 (84.5–100.0)
Lipoma	98.2 (95.7–100.0)	0.996 (0.986–1.000)	97.6 (94.7–100.0)	96.5 (91.7–100.0)	98.9 (97.4–100.0)
Pilomatricoma	94.5 (90.6–98.4)	0.905 (0.804–1.000)	78.8 (63.3–94.3)	75.9 (50.6–100.0)	97.4 (94.4–100.0)

^a^ Average of the values obtained for each class (epidermal cyst, lipoma, pilomatricoma). ^b^ Confidence Interval.

## Data Availability

S. Yang and B. Oh had full access to all data in the study and took responsibility for the data integrity and accuracy of the analysis. The datasets generated and analyzed during the current study cannot be publicly available due to patient privacy concerns but are available from the corresponding author upon reasonable request.

## Supplementary Materials

The following supporting information can be downloaded at: https://www.mdpi.com/article/10.3390/s23177374/s1, Figure S1: Overall flow of Fast AutoAugment; Figure S2: Joint distribution between the categorized confidence distribution and decision consistency; Figure S3: Confidence distribution and decision consistency distribution; Video S1: The demo video of web service for diagnosis; Table S1: Summary of sonographic features.

## References

[1] Wagner J.M., Lee K.S., Rosas H., Kliewer M.A. (2013). Accuracy of sonographic diagnosis of superficial masses. J. Ultrasound Med..

[2] Hwang E.J., Yoon H.S., Cho S., Park H.S. (2015). The diagnostic value of ultrasonography with 5-15-MHz probes in benign subcutaneous lesions. Int. J. Dermatol..

[3] Levy J., Barrett D.L., Harris N., Jeong J.J., Yang X., Chen S.C. (2021). High-frequency ultrasound in clinical dermatology: A review. Ultrasound J..

[4] Wortsman X. (2021). Practical applications of ultrasound in dermatology. Clin. Dermatol..

[5] Wortsman X. (2023). Top advances in dermatologic ultrasound. J. Ultrasound Med..

[6] Almuhanna N., Wortsman X., Wohlmuth-Wieser I., Kinoshita-Ise M., Alhusayen R. (2021). Overview of ultrasound imaging applications in dermatology. J. Cutan. Med. Surg..

[7] Oh B.H., Kim K.H., Chung K.Y. (2019). Skin imaging using ultrasound imaging, optical coherence tomography, confocal microscopy, and two-photon microscopy in cutaneous oncology. Front. Med..

[8] Quien M.M., Saric M. (2018). Ultrasound imaging artifacts: How to recognize them and how to avoid them. Echocardiography.

[9] Hoskins P.R., Martin K., Thrush A. (2019). Diagnostic Ultrasound: Physics and Equipment.

[10] Litjens G., Kooi T., Bejnordi B.E., Setio A.A.A., Ciompi F., Ghafoorian M., Van Der Laak J.A., Van Ginneken B., Sánchez C.I. (2017). A survey on deep learning in medical image analysis. Med. Image Anal..

[11] Yap J., Yolland W., Tschandl P. (2018). Multimodal skin lesion classification using deep learning. Exp. Dermatol..

[12] Li Y., Shen L. (2018). Skin lesion analysis towards melanoma detection using deep learning network. Sensors.

[13] Harangi B. (2018). Skin lesion classification with ensembles of deep convolutional neural networks. J. Biomed. Inform..

[14] Majtner T., Yildirim-Yayilgan S., Hardeberg J.Y. Combining deep learning and hand-crafted features for skin lesion classification. Proceedings of the 2016 Sixth International Conference on Image Processing Theory, Tools and Applications (IPTA).

[15] Yang X., Zeng Z., Yeo S.Y., Tan C., Tey H.L., Su Y. (2017). A novel multi-task deep learning model for skin lesion segmentation and classification. arXiv.

[16] Premaladha J., Ravichandran K. (2016). Novel approaches for diagnosing melanoma skin lesions through supervised and deep learning algorithms. J. Med. Syst..

[17] Czajkowska J., Juszczyk J., Piejko L., Glenc-Ambroży M. (2022). High-frequency ultrasound dataset for deep learning-based image quality assessment. Sensors.

[18] Bandari E., Beuzen T., Habashy L., Raza J., Yang X., Kapeluto J., Meneilly G., Madden K. (2022). Machine Learning Decision Support for Detecting Lipohypertrophy with Bedside Ultrasound: Proof-of-Concept Study. JMIR Form. Res..

[19] Kang S. (2019). Fitzpatrick’s Dermatology, 2-Volume Set (Fitzpatricks Dermatology in General Medicine).

[20] Oh B.H., Seo J., Chung K.Y. (2015). Surgical treatment of 846 patients with benign skin tumors: Experience of a dermatologic surgeon in Korea. Korean J. Dermatol..

[21] Zhou B., Khosla A., Lapedriza A., Oliva A., Torralba A. Learning deep features for discriminative localization. Proceedings of the IEEE Conference on Computer Vision and Pattern Recognition.

[22] Telea A. (2004). An image inpainting technique based on the fast marching method. J. Graph. Tools.

[23] Lim S., Kim I., Kim T., Kim C., Kim S. (2019). Fast autoaugment. arXiv.

[24] He K., Zhang X., Ren S., Sun J. Deep residual learning for image recognition. Proceedings of the IEEE Conference on Computer Vision and Pattern Recognition.

[25] Schlemper J., Oktay O., Chen L., Matthew J., Knight C., Kainz B., Glocker B., Rueckert D. (2018). Attention-gated networks for improving ultrasound scan plane detection. arXiv.

[26] Kingma D.P., Ba J. (2014). Adam: A method for stochastic optimization. arXiv.

[27] Wortsman X. (2012). Common applications of dermatologic sonography. J. Ultrasound Med..

[28] Lee H.S., Joo K.B., Song H.T., Kim Y.S., Park D.W., Park C.K., Lee W.M., Park Y.W., Koo J.H., Song S.Y. (2001). Relationship between sonographic and pathologic findings in epidermal inclusion cysts. J. Clin. Ultrasound.

[29] Wortsman X., Jemec G. (2013). Dermatologic Ultrasound with Clinical and Histologic Correlations.

[30] Chen C.Y., Chiou H.J., Chou S.Y., Chiou S.Y., Wang H.K., Chou Y.H., Chiang H.K. (2009). Computer-aided diagnosis of soft-tissue tumors using sonographic morphologic and texture features. Acad. Radiol..

[31] Chu Y.S., An H.G., Oh B.H., Yang S. (2020). Artificial Intelligence in Cutaneous Oncology. Front. Med..

[32] Cullell-Dalmau M., Noé S., Otero-Viñas M., Meić I., Manzo C. (2021). Convolutional neural network for skin lesion classification: Understanding the fundamentals through hands-on learning. Front. Med..

[33] Wells A., Patel S., Lee J.B., Motaparthi K. (2021). Artificial intelligence in dermatopathology: Diagnosis, education, and research. J. Cutan. Pathol..

[34] Haggenmüller S., Maron R.C., Hekler A., Utikal J.S., Barata C., Barnhill R.L., Beltraminelli H., Berking C., Betz-Stablein B., Blum A. (2021). Skin cancer classification via convolutional neural networks: Systematic review of studies involving human experts. Eur. J. Cancer.

[35] Ba W., Wu H., Chen W.W., Wang S.H., Zhang Z.Y., Wei X.J., Wang W.J., Yang L., Zhou D.M., Zhuang Y.X. (2022). Convolutional neural network assistance significantly improves dermatologists’ diagnosis of cutaneous tumours using clinical images. Eur. J. Cancer.

[36] Aggarwal P., Choi J., Sutaria N., Roh Y., Wongvibulsin S., Williams K., Huang A., Boozalis E., Le T., Chavda R. (2021). Clinical characteristics and disease burden in prurigo nodularis. Clin. Exp. Dermatol..

[37] Jartarkar S.R. (2023). Artificial intelligence: Its role in dermatopathology. Indian J. Dermatol. Venereol. Leprol..

[38] Lee S., Chu Y.S., Yoo S.K., Choi S., Choe S.J., Koh S.B., Chung K.Y., Xing L., Oh B., Yang S. (2020). Augmented decision-making for acral lentiginous melanoma detection using deep convolutional neural networks. J. Eur. Acad. Dermatol. Venereol..

[39] Yang Y., Wang J., Xie F., Liu J., Shu C., Wang Y., Zheng Y., Zhang H. (2021). A convolutional neural network trained with dermoscopic images of psoriasis performed on par with 230 dermatologists. Comput. Biol. Med..

[40] Kwiatkowska D., Kluska P., Reich A. (2021). Convolutional neural networks for the detection of malignant melanoma in dermoscopy images. Adv. Dermatol. Allergol. /Postępy Dermatol. I Alergol..

[41] Zhao W., Kang Q., Qian F., Li K., Zhu J., Ma B. (2022). Convolutional neural network-based computer-assisted diagnosis of Hashimoto’s thyroiditis on ultrasound. J. Clin. Endocrinol. Metab..

[42] Hu L., Pei C., Xie L., Liu Z., He N., Lv W. (2022). Convolutional Neural Network for predicting thyroid cancer based on ultrasound elastography image of perinodular region. Endocrinology.

[43] Nguyen T.N., Podkowa A.S., Park T.H., Miller R.J., Do M.N., Oelze M.L. (2021). Use of a convolutional neural network and quantitative ultrasound for diagnosis of fatty liver. Ultrasound Med. Biol..

[44] Liu J., Li W., Zhao N., Cao K., Yin Y., Song Q., Chen H., Gong X. Integrate domain knowledge in training CNN for ultrasonography breast cancer diagnosis. Proceedings of the International Conference on Medical Image Computing and Computer-Assisted Intervention.

[45] Zhou B.-Y., Wang L.-F., Yin H.-H., Wu T.-F., Ren T.-T., Peng C., Li D.-X., Shi H., Sun L.-P., Zhao C.-K. (2021). Decoding the molecular subtypes of breast cancer seen on multimodal ultrasound images using an assembled convolutional neural network model: A prospective and multicentre study. eBiomedicine.

[46] Lee J.H., Baek J.H., Kim J.H., Shim W.H., Chung S.R., Choi Y.J., Lee J.H. (2018). Deep Learning–Based Computer-Aided Diagnosis System for Localization and Diagnosis of Metastatic Lymph Nodes on Ultrasound: A Pilot Study. Thyroid.

[47] Xie B., Lei T., Wang N., Cai H., Xian J., He M., Zhang L., Xie H. (2020). Computer-aided diagnosis for fetal brain ultrasound images using deep convolutional neural networks. Int. J. Comput. Assist. Radiol. Surg..

[48] Born J., Wiedemann N., Brändle G., Buhre C., Rieck B., Borgwardt K. (2020). Accelerating COVID-19 differential diagnosis with explainable ultrasound image analysis. arXiv.

[49] Schneider S.L., Kohli I., Hamzavi I.H., Council M.L., Rossi A.M., Ozog D.M. (2019). Emerging imaging technologies in dermatology: Part II: Applications and limitations. J. Am. Acad. Dermatol..

