# Supplementary material for: Deep Learning-Based Evaluation of Ultrasound Images for Benign Skin Tumors
Source: Sensors (Basel). 2023 Aug 24;23(17):7374. doi: 10.3390/s23177374 (PMC10490539; doi:10.3390/s23177374)
Supplement: Supplementary file 1 [file sensors-23-07374-s001.zip › Supplementary Materials.pdf]

Supplementary

Figure S1. Overall flow of Fast AutoAugment

Figure S2. Joint distribution between the categorized confidence distribution and decision consistency

Figure S3. Confidence distribution and decision consistency distribution

Video S1. A demo video of web service for diagnosis

Table S1. Summary of sonographic features

Figure S1. Overall flow of Fast AutoAugment

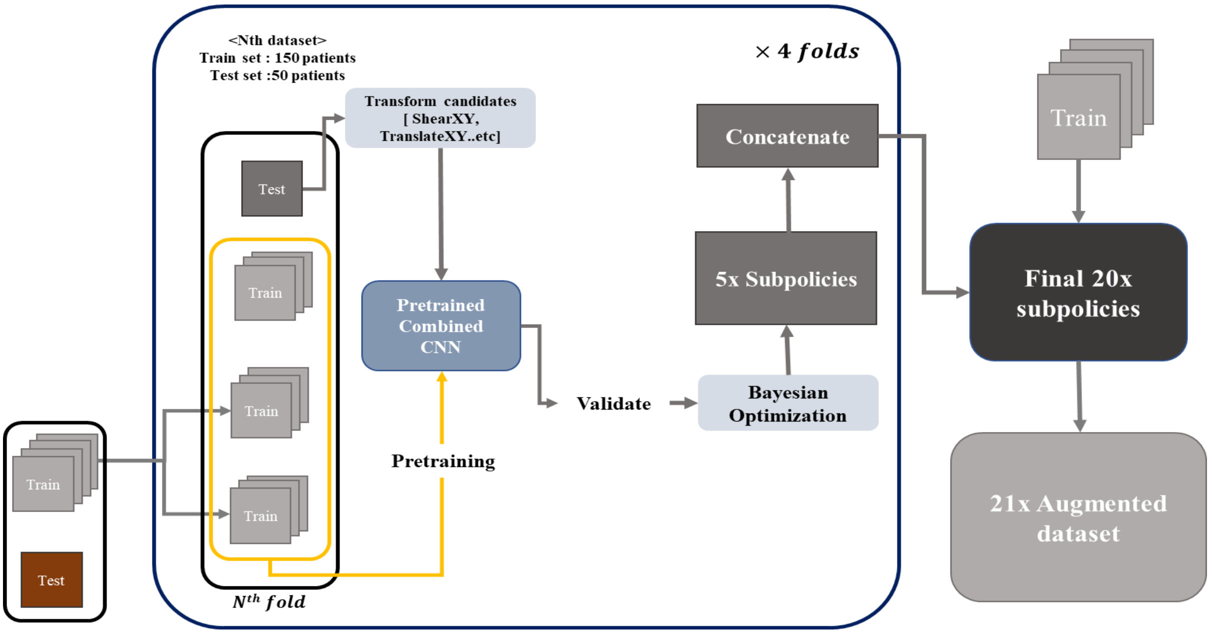

The figure above shows the overall process of the Fast AutoAugment algorithm used in this study. First, a total of 250 patients are divided into a train set and a test set in a 4:1 ratio. Then, after dividing the 200 patients in the train set into the train set and the test set once again 3:1, Combined CNN designed in this study is pretrained on the the re-separated train set. Then, two methods are randomly selected from transform candidates consisting of 14 methods such as shearing, auto contrasting, and cutout and one subpolicy is completed through the sequential probability and magnitude values of these two methods. The pretrained combined CNN is validated on the result of applying the subpolicy configured in this way to the test set, and Bayesian optimization is performed through the validation result. As a result, a probability distribution of model performance according to subpolicy is created, and 5 upper subpolicy is extracted according to the distribution. This whole process proceeds for 4 folds for the original train set, and subpolicies are concatenated for each fold, and finally, a total of 20 subpolicies are generated. Then, by applying the generated subpolicies to the original train set, finally 21 times augmentation is achieved.

Figure S2. Joint distribution between the categorized confidence distribution and decision consistency

Decision consistency (rows) × Confidence distribution (columns)

	0.4-0.6	0.6-0.8	0.8-1.0	Sum
Non-matched	1	2	1	4
Partial matched	8	15	40	63
All matched	27	60	544	631
sum	36	77	584	698

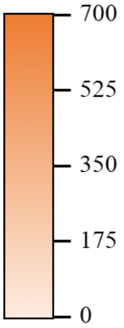

This table summarizes the joint distribution between the categorized confidence distribution and decision consistency.

Figure S3. Confidence distribution and decision consistency distribution

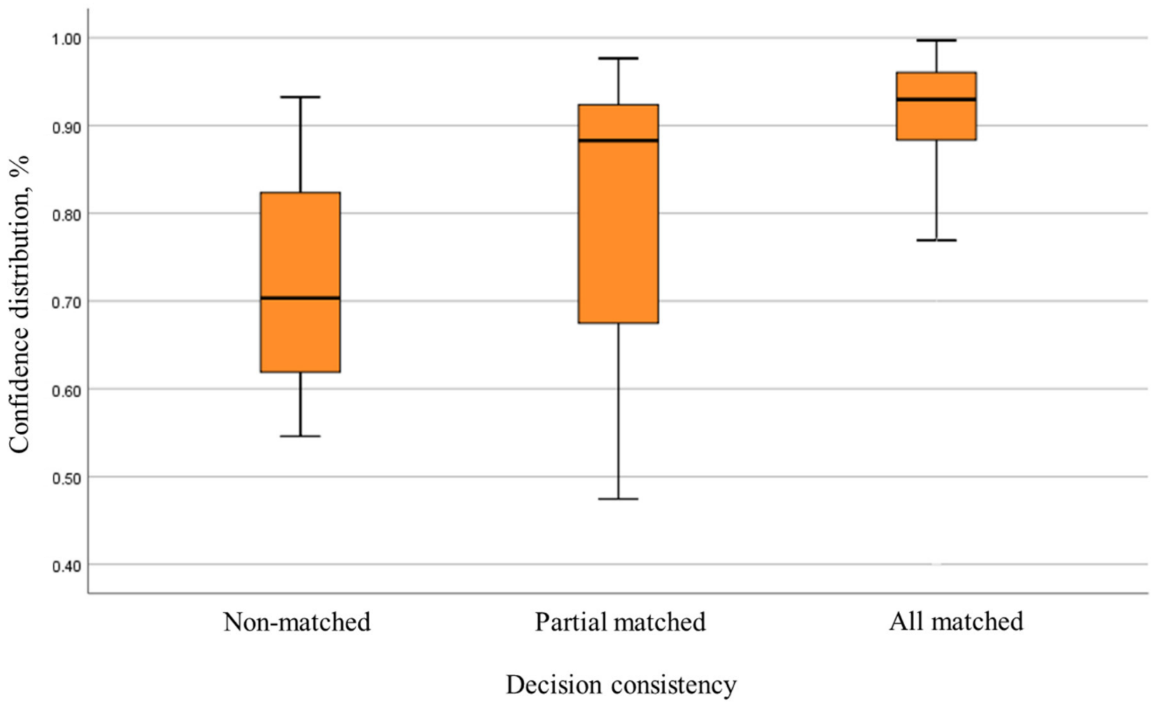

This is a box plot of the difference in the confidence distribution for each decision consistency level. Visually, it can be confirmed that there is some difference in confidence distribution according to the decision consistency level, and the actual ANOVA test obtains a significant result of a p-value of <0.001.

Video S1. The demo video of web service for diagnosis

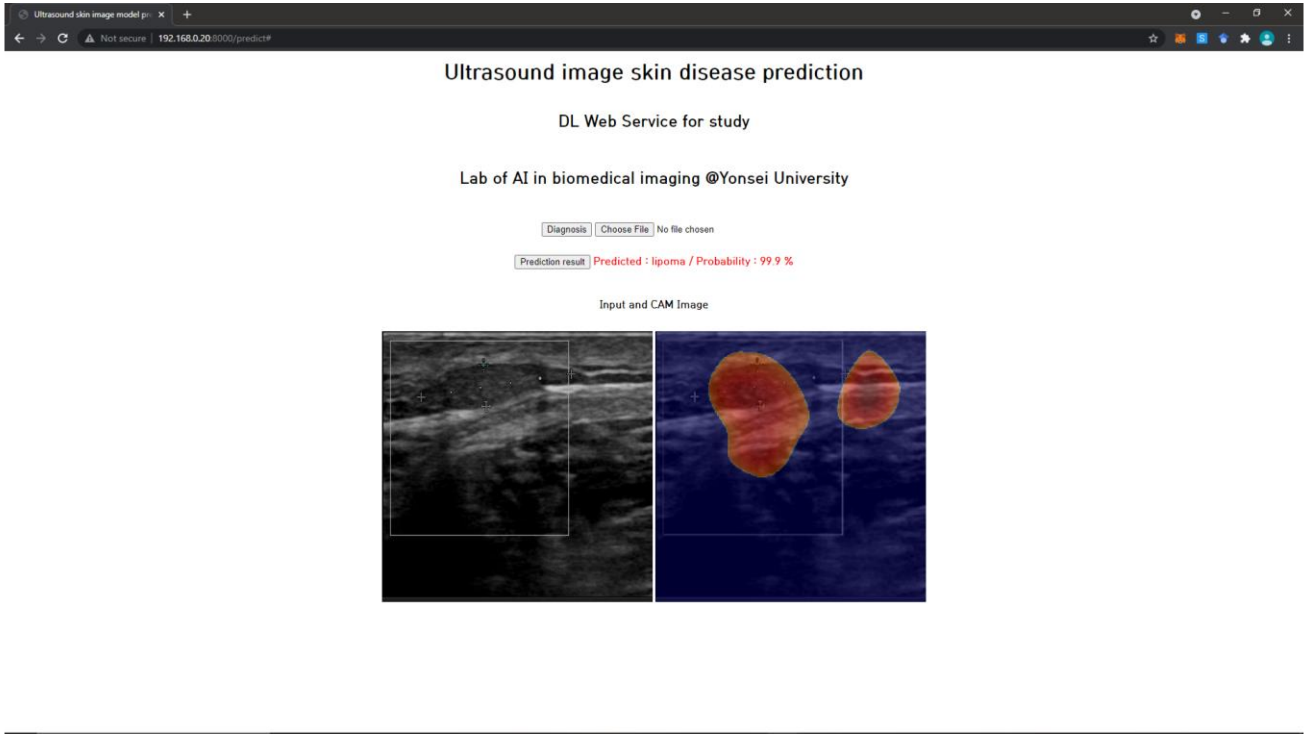

This is the demo video of the web service for the model trained in this study. After uploading the image file to be diagnosed and pressing the diagnosis button, the lesion name and probability predicted by the model are automatically displayed on the screen. In addition, the original image and the CAM image according to the model's prediction are displayed in the center of the screen.

Table S1. Summary of sonographic features (physicians' findings)

Diagnosis	Class	Sonographic Features (N, %)		Highlights of CAM		
				Totally Involved (N,%)	Partially Involved (N,%)	None (N,%)
**Epidermal cyst (N=388)**	**Intact (N=388)**	Well defined	379 (97.7)	374 (96.4)	14 (3.6)	0
		Hypoechoic	373 (96.1)			
		Anechoic	378 (97.4)			
		Posterior acoustic enhancement	384 (99.0)			
	**Ruptured (N=10)**	Ill defined	10 (100.0)	10 (100.0)	0	0
		Hypoechoic	10 (100.0)			
		Increased vascularity	5/7 (71.4)			
		Posterior acoustic enhancement	10 (100.0)			
		Increased echogenicity of the surrounding hypodermis	10 (100.0)			
**Lipoma (N=232)**	**Angiolipoma (N=80)**	Hyperechoic nodule	78 (97.5)	73 (91.3)	7 (8.8)	0
		Increased vascaularity	14/50 (28.0)			
	**Fibrolipoma (N=152)**	Hypoechoic with septae	152 (100.0)	127 (83.6)	24 (15.8)	1 (0.7)
**Pilomatricoma (N=68)**	**Targeted (N=35)**	Well defined	35 (100.0)	21 (60.0)	12 (34.3)	2 (5.7)
		Target	20 (57.1)			
		Acoustic shadowing	31 (88.6)			
		Well defined	20 (76.9)	16 (61.5)	9 (34.6)	1 (3.8)

	**Completely calcified (N=26)**	Strong posterior acoustic shadowing artifact	26 (100.0)			
	**Cystic (N=7)**	Well defined	7 (100.0)	7 (100.0)	0	0
		Cavity	7 (100.0)			

This table summarizes the sonographic features seen by experts (physicians' findings) and the data count for each feature and the degree to which each feature matches the class activation map (CAM).
